# Association of *TNP2* Gene Polymorphisms of the bta-miR-154 Target Site with the Semen Quality Traits of Chinese Holstein Bulls

**DOI:** 10.1371/journal.pone.0084355

**Published:** 2014-01-08

**Authors:** Qing Gao, Zhihua Ju, Yan Zhang, Jinming Huang, Xiaojian Zhang, Chao Qi, Jianbin Li, Jifeng Zhong, Guorong Li, Changfa Wang

**Affiliations:** 1 Dairy Cattle Research Center, Shandong Academy of Agricultural Science, Jinan, PR China; 2 College of Life Science, Shandong Normal University, Jinan, PR China; Wageningen UR Livestock Research, Netherlands

## Abstract

Transition protein 2 (TNP2) participates in removing nucleohistones and the initial condensation of spermatid nucleus during spermiogenesis. This study investigated the relationship between the variants of the bovine TNP2 gene and the semen quality traits of Chinese Holstein bulls. We detected three single nucleotide polymorphisms (SNPs) of the TNP2 gene in 392 Chinese Holstein bulls, namely, g.269 G>A (exon 1), g.480 C>T (intron 1), and g.1536 C>T (3′-UTR). Association analysis showed that the semen quality traits of the Chinese Holstein bulls was significantly affected by the three SNPs. The bulls with the haplotypic combinations H6H4, H6H6, and H6H8 had higher initial semen motility than those with the H7H8 and H8H4 haplotypic combinations (*P*<0.05). SNPs in the microRNA (miRNA) binding region of the TNP2 gene 3′-UTR may have contributed to the phenotypic differences. The phenotypic differences are caused by the altered expression of the miRNAs and their targets. Bioinformatics analysis predicted that the g.1536 C>T site in the TNP2 3′-UTR is located in the bta-miR-154 binding region. The quantitative real-time polymerase chain reaction results showed that the TNP2 mRNA relative expression in bulls with the CT and CC genotypes was significantly higher than those with the TT genotype (*P*<0.05) in the g.1536 C>T site. The luciferase assay also indicated that bta-miR-154 directly targets TNP2 in a murine Leydig cell tumor cell line. The SNP g.1536 C>T in the TNP2 3′-UTR, which altered the binding of TNP2 with bta-miR-154, was found to be associated with the semen quality traits of Chinese Holstein bulls.

## Introduction

The round spermatids of mammals undergo complex morphologic, physiologic, and biochemical modifications that result in the formation of mature spermatozoa. Major restructuring of the somatic chromatin occurs in elongating and condensing spermatids. Nucleohistones are first replaced by a group of arginine- and lysine-rich proteins called transition proteins (TNPs). The TNPs are then replaced by protamines [1], and the transcription stops. Then, the nucleosomal-type chromatin is transformed into a smooth fiber, and chromatin condensation begins [2].

At the transcriptional and the posttranscriptional levels, the haploid number during the differentiation phase of spermatogenesis in mammals is a striking system of developmental regulation of gene expression. Numerous new mRNAs are synthesized in early haploid cells into round spermatids. During the transcriptional inactivation of chromatin in late haploid cells, the round spermatids change into elongated spermatids [3]. mRNAs encode many of the proteins that are first synthesized during the final stage of sperm differentiation. The synthesized proteins are also transcribed in round spermatids. Before translation in elongated spermatids, the proteins are stored for five days in an inert state. Familiar examples include the mRNA of encoding transition proteins 1 and 2 (TNP1 and TNP2) and protamines 1 and 2 (PRM1 and PRM2) [4]. The genes PRM1, PRM2, and TNP2 are located on a 13/kb to 15 kb sequence in various species [5]. These genes exist as single, coordinately expressed genic domains. The gene order for humans, cattle, and pigs was found to be 5′-PRM1-PRM2-TNP2-3′ [6]–[8]. Previous analysis has shown that the genes within the human PRM1→PRM2→TNP2 domain are interrelated. Human genes share significant sequence similarity at both the nucleotide and amino acid levels [9], [10]. Reports have also shown the importance of delaying translation by demonstrating that premature translation of the TNP2 mRNAs in the round spermatids of transgenic mice impairs male fertility [11], [12].

miRNAs are small non-coding endogenous RNA molecules (18/nt to 24 nt). miRNAs mainly bind to partially complementary sequences in the 3′-untranslated region (UTR) to inhibit the target mRNA translation into protein or accelerate mRNA degradation [13], [14]. A study has proven that miRNAs participate in regulating the quality of semen [15]. Recent studies have also suggested that miRNA-154 is differentially expressed in immature and mature testicular tissues of humans and rhesus monkeys [16]. During primate spermatogenesis, the miRNA-154 also regulates the expression of a series of genes that are essential for the formation and differentiation of different types of cells (mainly spermatocytes and spermatids). Bioinformatics prediction showed that the bovine TNP2 gene is targeted by bta-miR-154. SNPs associated with polygenetic disease create, destroy, or modify miRNA binding sites [17]. However, the extent to which the SNPs of the TNP2 gene interfere with miRNA gene regulation and affect semen quality traits are unknown.

The present study attempts to contribute in overcoming disturbances by identifying potentially expressed candidate genes and studying the association in male gametes and in reproductive tissues in bulls. This study also focuses on the association of TNP2 gene with the semen quality traits in Chinese Holstein bulls and the effects of testicular miRNA on TNP2 gene expression.

## Materials and Methods

### Animals and DNA extraction

The association of SNPs with semen quality traits (e.g., ejaculate volume, initial sperm motility, sperm density, post-thaw cryopreserved sperm motility, and deformity rate) was analyzed using 392 Chinese Holstein bulls from three bull stations (Beijing Dairy Center, Shanghai Bright Dairy and Food Co. Ltd, and Shandong OX Bio-Technology Co. Ltd). All of collected semen samples were collected by company workers with permission from the animal owners. This study was approved by the Chinese Ministry of Agriculture Bureau of Animal Husbandry and Veterinary and the Ministry of Agriculture Dairy Cattle Frozen Semen Quality Supervision Testing Center.

The semen quality traits of each ejaculate sample were determined under light microscopy according to the guidelines provided by the World Health Organization. The sperm samples were labeled with the date and the age of the bulls. The semen quality traits of each bull were repeatedly measured from 2008 to 2012. Data on 39125 ejaculate samples from the 392 bulls were used, with 4 to 379 ejaculate samples from each bull. The semen quality traits were averaged for each bull using the mean function of the SAS Software. Genomic DNA was extracted from the sperm samples using standard protocols. The samples were treated with mercaptoethanol and digested with proteinase K, followed by salt extraction and isopropanol precipitation. DNA content was spectrophotometrically estimated and the mixture was diluted to 50 ng/µL. All DNA samples were stored at −20°C for subsequent analysis.

### SNP detection and genotyping

Primers for detecting polymorphism and amplifying the bull TNP2 gene (GenBank accession no. NC_007326.4) (Table 1) were designed using PRIMER PREMIER 5.0 software (Premier, Canada). Polymerase chain reaction (PCR) was carried out using 0.5 U of Taq DNA polymerase (TaKaRa, Dalian, China), 2.5 µL of 10× PCR Buffer, 2.5 mmol/L MgCl_2_, 0.2 mmol/L dNTPs, 0.5 µmol/L of each primer, and 50 ng DNA in a total volume of 25 µL. After an initial denaturation for 5 min at 95°C, PCR was performed with 35 cycles of denaturation at 95°C for 30 s; annealing at 54°C to 62°C (Table 1) for 30 s; elongation at 72°C for 30 s. The final extension was performed at 72°C for 10 min. PCR was carried out using a PCR System Thermal Cycler Dice (TaKaRa, Dalian, China). The PCR product was electrophoresed on 1% agarose gel and directly sequenced using an ABI PRISM™ 3730 DNA sequencer (Applied Biosystems) and a BigDye terminator v3.1 sequencing kit (Shanghai Sangon, China). Sequence data were analyzed by detecting polymorphisms using the DNASTAR 5.0 package (DNASTAR, Inc., USA).

**Table 1:** PCR primers and PCR-RFLP tests for genotyping in the bovine TNP2 gene.

SNP	Primer sequences	AT (°C)	SAF (bp)/AR	RE	RES, bp/genotype
g.269 G>A	F: CAGAGCCTTCCCAACACCC	62	195(100–294)	*Hpa*II	GG: 135, 33, 27
	R: GCTGGGGCCTGGGCTCTGG				GA: 135, 60, 33, 27
					AA: 135, 60
g.480 C>T	F: AGGAGCCACCGCAGCCCCACTGGGC	59	350(244–593)	*Hpa*II	CC: 237, 113
	R: TGCCTGTGGGTCCTCTGTGC				CT: 350, 237, 113
					TT: 350
g.1536 C>T	F: ACTGGACCAATGAACGAA	54	535(1101–1635)	*Hin*dIII	CC: 535
	R: CTCCCTACCCAACCTCTT				CT: 535, 432, 103
					TT: 432, 103

Note: Underlined nucleotides mark nucleotide mismatches enabling the use of the selected restriction enzymes for discriminating sequence variations. AT annealing temperature, SAF size of amplification fragment, RE restriction enzyme, and RES size of fragments at the indicated allele after digestion of the PCR product use the respective restriction enzyme and SNP single nucleotide polymorphism.

The three SNPs of the TNP2 gene (g.269 G>A; g.480 C>T; g.1536 C>T, translated start point is ATG as +1) were genotyped according to the sequencing result using PCR Restriction Fragment Length Polymorphism and Created Restriction Site PCR (CRS-PCR). The PCR products were cleaved using *Hpa*II and *Hin*dIII. The g.269 G>A polymorphism was genotyped via the CRS-PCR method with one of the primers containing a nucleotide mismatch. The CRS-PCR also enabled the use of the restriction enzyme *Hpa*II for discriminating sequence variations [18]. Aliquots of the PCR products (1 µL) were digested with 10 U of *Hpa*II at 37°C for 30 min. The digested products were detected via electrophoresis in 10% PAGE (29 acrylamide∶1 bisacrylamide) in 1× TBE buffer and constant voltage (110 V) for 3.5 h at room temperature. The digested products were then stained with 0.1% silver nitrate [19]. The primers, restriction enzymes, and fragment sizes are given in Table 1. The results of allelic variation assay at the SNP sites were based on the electrophoretic pattern of the restriction enzyme-treated PCR products.

### Bioinformatics analysis

We performed MTar searches of the putative miRNA-binding sites of the 3′ UTRs from segments of perfect Watson-Crick complementarity to bases 2–8 of the miRNA numbered from the 5′ end [20]. Comparative sequence analysis revealed one SNP, g.1536 C>T, was in the bovine TNP2 gene 3′-UTR fragment. To identify the putative miRNA-binding sites of the bovine TNP2 gene 3′ UTRs, we integrated the output results of three prediction programs: Targetscan (http://www.targetscan.org/vert_50/), MicroInspector (http://bio2server.bioinfo.uni-plovdiv.bg/microinspector/), and RNA22 microRNA target detection (http://cbcsrv.watson.ibm.com/rna22.html) [21], [22].

### Quantitative miRNA analysis

RNA was extracted from the bull testes to compare the differential expression of bta-miR-154 between adult [2.5 years old (post-sex maturation)] and fetal bulls [3 days old (pre-sex maturation)]. An miScript Reverse Transcription Kit (Qiagen) was used for cDNA synthesis to detect miRNA expression, and an miScript SYBR Green PCR Kit (Qiagen) with a bta-miR-154 specific primer was used to detect mature miRNA detection. Bta-let-7g was used as the internal control. Relative gene expression was determined using a previously described method [23]. The miRNA from each sample was analyzed in triplicate. The relative miRNA quantity was represented as fold changes. Student's t-tests were used to determine the significance of the fold changes.

### Measurement of the expression of TNP2 mRNA

Samples were collected from the testicular tissues of nine bulls from a commercial slaughter farm. Total RNA was extracted from the testicular tissues samples using TRIzol reagent (Invitrogen) according to the manufacturer's protocol. The relative expression of TNP2 mRNA in the testicular tissues of bulls with different genotypes in the SNP (g.1536 C>T) locus was determined via quantitative real-time PCR (qPCR) using the primer (F: 5′-AAGCCACGCCTGCCACCACT-3′; R:5′-TCCCTCCAAGTTCTTTCTGTTC-3′; product size = 247 bp). The primers of housekeeping internal control β-actin gene were designed according to reference [21]. qPCR was performed using an SYBR green assay (TaKaRa Biotechnology, Dalian, China) on a Roche LightCycler 480 machine (Roche Applied Science, Mannheim, Germany). The qPCR was performed as follows: 50°C for 2 min; 94°C for 3 min; followed by 40 cycles of 94°C for 30 s, 55°C for 40 s, 68°C for 15 s. The last stage for the dissociation curve was as follows: 95°C for 15 s; 60°C for 15 s; and 95°C for 15 s. All reactions were done in a 20 µL reaction volume in triplicate.

### Construction of 3′-UTR-luciferase expression plasmid

The TNP2 3′-UTR was amplified using a pair of primers with the following sequences 5′-CGACGCGTATAAGAAACGCCATCAC-3′ and 5′-CCCAAGCTTACCTCTTCCCTGCTTTGT-3′ to produce luciferase constructs containing g.1536 C>T. The PCR was performed using genomic DNA from subjects homozygous for the 1536 CC gene. The TT genotype was also used to generate 1536 C and 1536 T luciferase constructs. The PCR product covering the seed sequence region of bta-miR-154 that binds to TNP2 3′-UTR was digested using *Mlu* I/*Hin*d III and ligated into pMIR-REPORT vector (Promega). Cloning was performed and used to transform Trans5α cells (Invitrogen). Cloning was also plated on agar containing 100 mg/mL ampicillin and incubated overnight at 37°C. Positive colonies were cultured in 10 mL of lysogeny broth medium (Fisher BioReagents) containing 100 mg/mL ampicillin and incubated overnight at 37°C. Plasmids were isolated using a Plasmid Miniprep Kit (Biomiga) according to the manufacturer's instructions. The plasmid DNA was quantified using a NanoDrop ND-1000. The constructs were sequenced to ensure proper orientation and authenticity in the vector.

### Cell culture and treatments

A murine Leydig cell tumor cell line (MLTC-1) was purchased from the Shanghai Life Science Institute, Chinese Academy of Sciences Cell Resource Center. MLTC-1 was maintained in RPMI 1640 supplemented with 1.5 g/L sodium bicarbonate, 4.5 g/L glucose, 10 mM HEPES, 1.0 mM sodium pyruvate, 2 mM L-glutamine, 500 U/L penicillin, 500 mg/L streptomycin, and 10% (v/v) fetal bovine serum (FBS) [24]–[25]. The cells were maintained at 37°C with 5% CO_2_ and subcultured every other day. All reagents were from GIBCO unless otherwise specified.

CCS-bta-154-MR04 (the precursor sequence of bta-miR-154: gcggaacuugaagauagguuauccguguagccuucgcuuuacuugugacgaaucauacacgguucaccuauuuuuuaguaccaa) and the scrambled control miRNA (CmiR0001-MR04) were purchased from GeneCopoeia.

### Transient transfection and luciferase reporter assay

The cells were distributed into a 48-well culture tray 1 day before the transient transfection assay. The culture tray contained 3 mL of RPMI 1640 complete media per well after subculturing. Upon reaching 80% to 90% confluence, the cells were transfected with 200 ng of the luciferase expression constructs and 100 ng to 400 ng of miR-154 or 200 ng of the control vector without miRNA insert using Lipofectamine 2000 (Invitrogen, Carlsbad, CA, USA). The plasmids were cotransfected with 40 ng of β-gal for normalization. All experiments were performed with a negative control plasmid (pMIR-REPORT™). About 48 h after transfection, the cells were washed once with phosphate-buffered saline before being photographed. The cells were also lysed once with 65 µL of reporter lysis buffer per well (Promega). The cell lysates from the transfected cells were prepared and assayed according to the manufacturer's instructions (Promega) for both firefly luciferase and β-gal absorbance assays [26]. All transfection data are representative of six independent transfections using at least two independent preparations of both DNA and plasmid clones. Activity was expressed relative to firefly luciferase activity normalized against β-gal absorbance at a of wavelength 405 nm [24].

### Statistical analysis

The SHEsis software (http://analysis.bio-x.cn) was used to analyze the pairwise linkage disequilibrium and haplotypic frequencies [27]. The associations between the TNP2 gene SNPs and semen quality traits were analyzed using a general least-square model procedure using statistical analysis software (SAS Institute Inc., Cary, NC, USA, 2002). The linear model is expressed as follows:
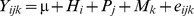
where *Y* *_ijk_* is the observed value of each semen quality trait; *μ* is the overall mean; *H_i_* is the fixed effect of genotypic or haplotypic combinations; *P_j_* is the fixed effect of age (*j* = 2–10; classified as: (1) 2–3 years; (2) 4–5 years; (3) 6–10 years); *M_k_* is the effect of farm; *e_ijk_* is the random residual error. Values with *P*<0.05 and *P*<0.01 were considered significant. Multiple comparisons were performed using Duncan's method.

The effect of bta-miR-154 on TNP2 activity was tested using a one-way ANOVA. Values are represented as means ± standard error of the mean (SEM).

## Results

### Genetic polymorphisms of TNP2 gene in Chinese Holstein bulls

**Figure 1:**
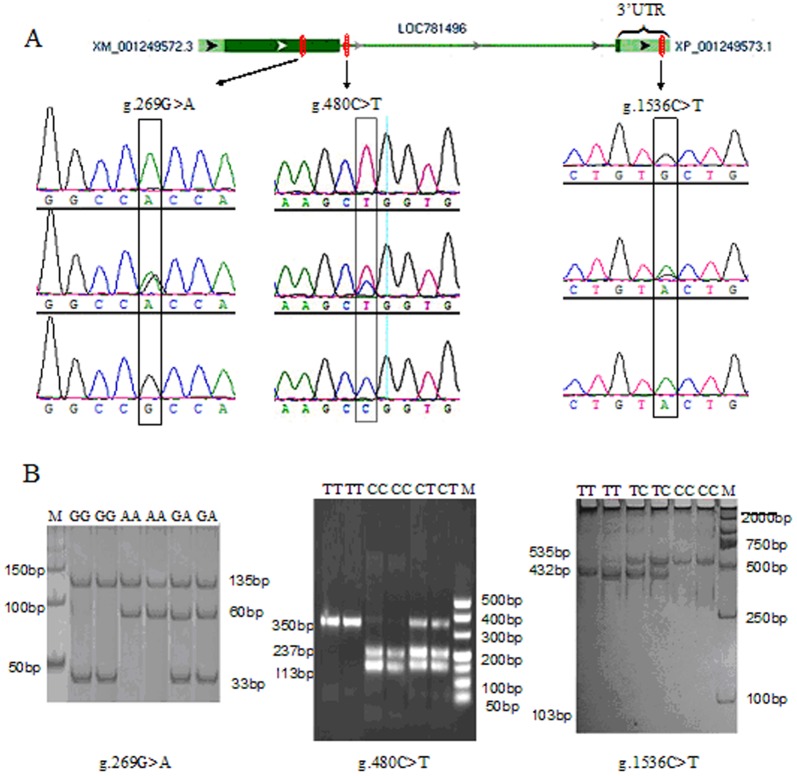
TNP2 gene structure, SNP location, sequencing results, and band patterns of genotypes g.269G>A, g.480C>T, and g.1536C>T. Part A shows the bovine TNP2 gene structure, location, and SNP sequencing results. The green region represents the exon and blue region represents the untranslated region (UTR). B: Silver-stained gels showing the band patterns of SNPs g.269G>A, g.480C>T, and g.1536C>T digested with *Hpa*II and *Hin*dIII. *Hpa*II digestion of the PCR products of the TNP2 g.269G>A locus produced 135, 33, and 27 bp bands for the GG genotype; 135, 60, 33, and 27 bp bands for the GA genotype; 135 and 60 bp bands for the AA genotype. *Hpa*II digestion of the PCR products of the TNP2 g.480C>T locus resulted in a 350 bp band for the TT genotype; 350, 237, and 113 bp bands for the CT genotype; 237 and 113 bp bands for the CC genotype. *Hin*dIII digestion of the PCR products of the TNP2 g.1536 C>T locus generated 432 and 103 bp bands for the TT genotype; 535, 432, and 103 bp bands for the TC genotype; a 535 bp band for the CC genotype.

**Figure 2:**
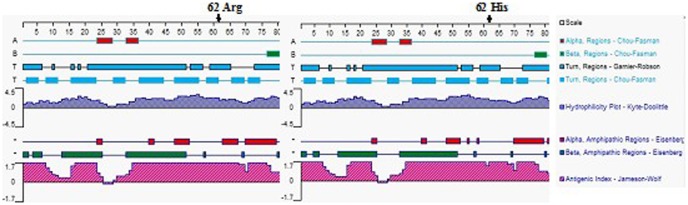
Prediction of the secondary structure of TNP2 and analysis of its hydrophilicity. The Arg→His substitution at position 62 change the hydrophilicity and antigenic index of TNP2.

**Figure 3:**
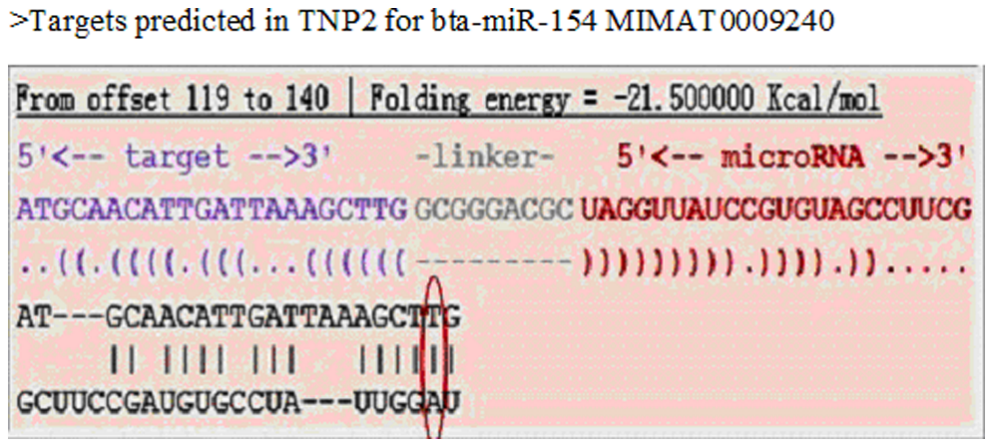
The single nucleotide polymorphism g. 1536 C>T located in the bta-miR-154 seed region binds to the 3′-UTR of the bovine TNP2 gene.

Three SNPs were revealed from 40 randomly selected samples, namely, g.269 G>A (exon 1), g.480 C>T (intron 1), and g.1536 C>T (3′-UTR). Direct sequencing and comparisons with the reference sequence (NC_007326.4) were used to select the 40 samples (Fig. 1). The SNP g.269 G>A was identified as a non-synonymous mutation [CGC (Arg)>CAC (His)] at position 62 of TNP2 (132 aa). The hydrophobicity and antigenic index of the protein changed in the SNP g.269 G>A located in the middle region of the protein when the amino acid also changed (Fig. 2). The non-synonymous SNP g.269 G>A may have caused the changes in the secondary structure of TNP2, which leads to changes in protein function. We also found that SNP g.1536 C>T is located within the 3′-UTR of the TNP2 gene. According to the three different systemic bioinformatics software that we used, TNP2 is predicted one of the target genes of bta-miR-154. The SNP g.1536 C>T was located within the bta-miR-154 binding site (Fig. 3).

Locus g.269 G>A was genotyped by employing CRS-PCR method with an antisense primer containing a nucleotide mismatch. The method enables *Hpa*II to discriminate sequence variations. *Hpa*II digestion of the PCR products of the TNP2 g.269 G>A locus produced the following: 135-, 33-, and 27-bp fragments for genotype GG; 135-, 60-, 33-, and 27-bp fragments for genotype GA; and 135- and 60-bp fragments for genotype AA. *Hpa*II digestion of the PCR products of TNP2 g.480 C>T locus produced the following: 350-bp fragments for genotype TT; 350-, 237-, and 113-bp fragments for genotype TC; and 237- and 113-bp fragments for genotype CC. *Hin*dIII digestion of the PCR products of TNP2 g.1536 C>T locus produced the following: 432- and 103-bp fragments for genotype TT; 535-, 432-, and 103-bp fragments for genotype TC; and 535-bp fragments for genotype CC (Fig. 1).

Table 2 shows the allelic and genotypic frequencies, the *χ^2^* values, the heterozygosities (*H_e_*), and the other genetic indices of the three SNPs of the bovine TNP2 gene. Alleles *G*, *C*, and *T* were the dominant alleles at positions g.269 G>A, g.480 C>T, and g.1536 C>T in the bulls, respectively. At locus g.480 C>T, the frequencies of genotype CC were higher than those of the genotype TT. The locus possessed low genetic diversity (*P*<0.25). The Chinese Holstein bulls possessed intermediate genetic diversity at the g.269 G>A and g.1536 C>T loci (0.25<*P*<0.50), which suggests intermediate genetic diversity. The results of the χ^2^ test showed that all SNPs were inconsistent with the Hardy–Weinberg equilibrium (Table 2). The selection pressure on the three SNPs was powerful. The linkage disequilibrium between the three SNPs in the population indicated that the SNPs were unlinked.

**Table 2:** Genotypic and allelic frequencies and Hardy–Weinberg equilibrium χ^2^ test of TNP2 gene at positions: g.269 G>A, g.480 C>T and g.1536 C>T.

Locus	Genotype	Genotypic frequency (Number)	Allele	Allelic frequency	*PIC*	*He*	*Ne*	*χ^2^*(*P* value)
g.269 G>A	GG	0.666(261)	G	0.759	0.299	0.366	1.577	94.530(2.414E^−22^)
	GA	0.186(73)	A	0.241				
	AA	0.148(58)						
g.480 C>T	CC	0.821(322)	C	0.884	0.184	0.205	1.258	59.878(1.009E^−11^)
	CT	0.125(49)	T	0.116				
	TT	0.054(21)						
g.1536 C>T	CC	0.158(62)	C	0.357	0.354	0.459	1.849	6.969(0.00829)
	CT	0.398(156)	T	0.643				
	TT	0.444(174)						

Note: *He* = heterozygosity; *Ne* = effective of alleles; *PIC* = polymorphism information content.

### Association of TNP2 gene polymorphism with the semen quality traits of Chinese Holstein bulls

We analyzed the association of the three SNPs with semen quality traits (Table 3). We included ejaculate volume, initial sperm motility, sperm density, post-thaw cryopreserved sperm motility, and deformity rate in Chinese Holstein bulls. The bulls with genotype GA at locus g.269 G>A had lower initial sperm motility and higher deformity rates than those with genotypes GG and AA (*P*<0.05). Our results show that cows with genotype TT at locus g.480 C>T had lower deformity rates (*P*<0.05) than those with genotypes CC and CT. Bulls with genotype CT at locus g.1536 C>T had higher ejaculate volumes and post-thaw cryopreserved sperm motility than those with genotype CC (*P*<0.05).

**Table 3:** Least squares mean and standard errors of semen quality traits of 392 Chinese Holstein bulls with different TNP2 genotypes.

Loci	Genotype(Number)	Ejaculate volume (mL)	Initial sperm motility (%)	Sperm density (×10^8^/mL)	Post-thaw cryopreserved sperm motility (%)	Deformity rate (%)
g.269 G>A	GG(261)	6.16±0.11	73.55±0.44^a^	10.47±0.17	40.39±0.40	13.55±0.28^b^
	GA(73)	6.43±0.26	71.45±1.04^b^	10.19±0.40	40.21±0.92	15.99±0.47^a^
	AA(58)	6.31±0.20	74.06±0.78^a^	11.17±0.30	39.84±0.69	13.08±0.44^b^
g.480 C>T	CC(322)	6.21±0.10	73.33±0.39	10.48±0.15	38.59±0.35	14.13±0.23^a^
	CT(49)	6.24±0.22	73.24±0.88	10.75±0.34	40.20±0.78	14.47±0.60^a^
	TT(21)	6.78±0.73	74.13±2.88	11.30±1.10	40.46±2.53	12.57±1.62^b^
g.1536 C>T	CC(62)	6.00±0.47^b^	74.34±1.86	10.67±0.72	37.28±1.63^b^	13.92±0.89
	CT(156)	6.94±0.38^a^	73.73±1.52	10.67±0.59	41.23±1.32^a^	14.31±0.89
	TT(174)	6.23±0.09^ab^	73.29±0.38	10.56±0.15	40.10±0.34^ab^	14.77±0.23

**Notes**: Means with the different lowercase letters within the same column are significantly different (*P*<0.05).

The SNPs g.269 G>A, g.480 C>T, and g.1536 C>T were used for haplotype reconstruction. The haplotypes were H1 (ACC), H2 (ACT), H3 (ATC), H4 (ATT), H5 (GCC), H6 (GCT), H7 (GTC), and H8 (GTT). The estimated haplotypic frequencies were 1.9%, 4.6%, 0.7%, 1.8%, 25.2%, 47.3%, 4.9%, and 13.6%, respectively. H6 had the highest haplotypic frequency, whereas H3 had the lowest. The correlation among haplotypic combinations and semen quality traits was analyzed (Table 4) by combining H6 and H#. Eighteen haplotypic combinations of TNP2 were detected from the tested bulls. The number of bulls with haplotypic combinations H1H2 (AACCCT/4), H2H2 (AACCTT/3), H1H3 (AACTCC/1), H4H4 (AATTTT/2), H5H4 (AGCTCT/3), and H7H4 (AGTTCT/4) was less than 5, which is not statistically significant. The association between the haplotypic combinations and the semen quality traits of Chinese Holstein bulls was not analyzed.

**Table 4:** Effect of the different combinations of the SNPs g.269 G>A, g.480C>T, and g.1536C>T on the semen quality traits of Chinese Holstein bulls.

Combined haplotypes	Sample size	Ejaculate volume (mL)	Initial sperm motility (%)	Sperm density (×10^8^/mL)	Post-thaw cryopreserved sperm motility (%)	Deformity rate (%)
H1H4	7	6.47±0.25	72.65±0.99	14.71±0.37^a^	42.60±0.82	11.86±0.72^a^
H5H1	19	6.66±0.39	70.71±1.57	11.22±0.59	35.53±1.27	14.34±0.96
H5H2	42	5.60±0.57	68.80±1.30	9.36±0.86^b^	38.14±1.86	15.22±1.57
H6H2	17	5.84±0.52	66.83±2.06	10.08±0.77	40.97±1.67	14.89±0.86
H6H4	8	6.17±0.61	64.65±1.45^b^	9.48±0.92^b^	40.19±1.99	14.45±1.11
H8H4	21	7.77±0.12	74.87±0.49^a^	11.39±0.18	41.34±0.40	12.54±0.30
H5H6	84	5.99±0.27	70.19±1.03	10.36±0.41	40.61±0.88	14.40±0.72
H6H6	36	6.65±0.36	64.74±1.43^b^	12.24±0.54	37.78±1.19	17.11±0.64^b^
H5H8	57	6.65±0.43	72.17±1.44	10.26±0.64	35.26±1.47	16.40±0.62
H6H8	16	6.29±0.41	61.92±0.85^b^	13.01±0.57	31.84±0.91	19.47±0.93^b^
H7H8	25	6.98±0.28	74.66±0.81^a^	11.75±0.48	41.14±1.57	12.24±0.47^a^
H8H8	43	6.14±0.40	65.19±1.09	10.26±0.82	34.78±1.41	13.40±0.42

Note: Means with the same small letters within the same row differ at *P*<0.05. Means marked with different superscript or without any superscript do not differ statistically. H1 = ACC, H2 = ACT, H3 = ATC, H4 = ATT, H5 = GCC, H6 = GCT, H7 = GTC, and H8 = GTT.

Statistical analysis showed that initial sperm motility, sperm density, and deformity rate significantly differed (*P*<0.05) among the different haplotypic combinations (three SNPs) (Table 4). No significant differences in ejaculate volume and post-thaw cryopreserved sperm motility were observed between the various haplotypic combinations. The bulls with haplotypic combinations H8H4 and H7H8 showed a higher initial sperm motility (*P*<0.05) than those with H6H4, H6H6, and H6H8 (*P*<0.05). The bulls with the haplotypic combination H1H4 had significantly higher sperm densities than those with H5H2 and H6H4 (*P*<0.05). The bulls with haplotypic combinations H1H4 and H7H8 showed significantly lower deformity rates than those with H6H6 and H6H8 (*P*<0.05).

### SNP g.1536 C>T is located within miRNA-154 binding site in TNP2 3′-UTR

We identified miRNAs that can potentially regulate TNP2 expression with one SNP present in the miRNA seed sequence. To identify the miRNA, we computationally predicted which miRNAs might contribute to TNP2 regulation. We used different algorithms to reduce the false positive rate of the target prediction results. We selected miRNA target pairs with context score of 0.10 or above from the possible putative sites. The miRNA target pairs were predicted to bind with the same target site by all the three software. The miRNAs that were then predicted by at least 2 of the algorithms to bind were considered candidates for further study. We integrated the results from the three prediction software programs. The prediction results showed that the SNP g.1536 C>T of the 3′-UTRs, which alters the binding to the bta-miR-154 seed sequence, may regulate TNP2 gene expression. Only bta-miR-154 had a high likelihood for targeting TNP2 3′-UTR with g.1536 C>T-T (Fig. 3). TNP2 binding was not observed in the g.1536 C>T-C SNP mutation.

### Expression of bta-miR-154 in the testis tissues of bulls

Huang et al. (2011) discovered the novel miRNA miR-154 in cows through Solexa sequencing. They detected the differential expression between testicular and ovarian tissues [23]. The results show that bta-miR-154 expression is male specific. In the present study, we detected the relative quantities of bta-miR-154 in the testicular tissues of adult and fetal bulls. The results suggest that bta-miR-154 expression is upregulated 1.4-fold in adult bull testicular tissues compared with the fetal bull testicular tissues.

### SNP g.1536 C>T affects TNP2 mRNA expression

**Figure 4:**
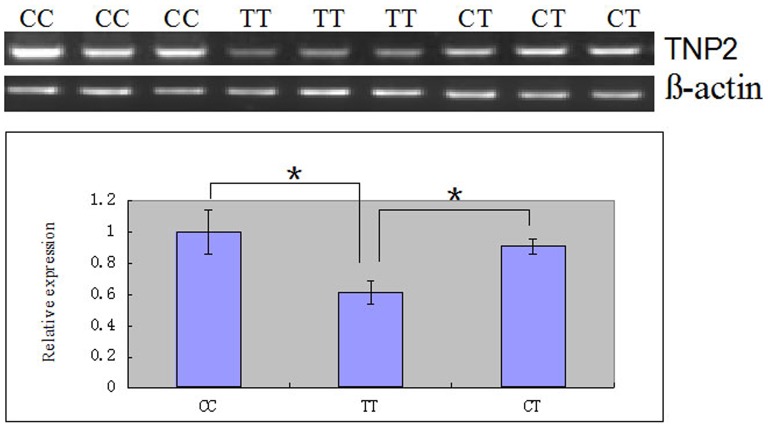
Relative TNP2 mRNA expression in bull testicles with genotypes CC, CT, and TT in the SNP g. 1536 C>T locus. The bovine β-actin gene was the housekeeping used as the internal control. The relative TNP2 mRNA expression is presented as mean ± SEM; vertical bars denote the SEM.

The TNP2 mRNA levels in the testicles of bulls with different genotypes were determined to investigate the effect of SNP g.1536 C>T on TNP2 expression. We found that the expression of TNP2 mRNA with genotypes CT and CC was significantly higher than that with genotype TT (*P*<0.05; Fig. 4). The high TNP2 expression is conducive to spermatogenesis and consistent with the results of the association analysis. The results showed that the SNP g.1536 C>T in the TNP2 3′-UTR affected TNP2 mRNA expression.

### Effect of SNP g.1536 C>T on bta-miR-154 targeting

**Figure 5:**
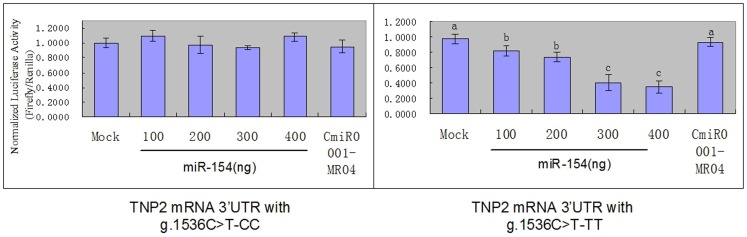
miR-154 directly binds to the 3′-UTR of the mutant TNP2 mRNA. A luciferase reporter vector containing the TNP2 mRNA 3′-UTR was cotransfected with miR-154 in MLTC-1 cells. The left chart indicates the luciferase activity before the mutation of locus g.1536 C>T. The right chart represents the results after the mutation. Bta-miR-154 at concentrations of 100/ng to 400 ng dose-dependently repressed the reporter activity. Mock transfected cells were treated with the luciferase reporter vector alone. The values are shown as means ± SEM (n = 3). Random nucleotides served as the negative control. Means without a common letter have significant differences (*P*<0.05).

A bovine TNP2 3′-UTR fragment containing either the wild-type or the mutant miR-154-binding sequence was subcloned downstream of the luciferase expression vector to confirm that miR-154 targets TNP2. The reporter containing the g.1536 C>T-CC TNP2 3′-UTR did not significant influence miR-154 expression and the luciferase activity No regardless of the concentration (*P*>0.05). By contrast, increasing the miR-154 concentrations in the g.1536 C>T-TT samples dose-dependently repressed the luciferase activity (*P*<0.05). Figure 5 shows the cotransfected cells with luciferase reporter vector. The results indicate direct binding between miR-154 and the cloned TNP2 mRNA 3′-UTR sequence, with miR-154 inhibiting the upstream luciferase gene. The repression was dependent on TNP2 mRNA 3′-UTR because miR-154 did not affect the luciferase reporter activity of the pMIR-control vector (Fig. 5). Random nucleotides served as the negative control for miR-154 transfections. The results indicate that 1536 C-T mutation caused the target binding of the microRNA. The target binding resulted in transcriptional repression and induced expression.

## Discussion

TNP2 is located on chromosome 25 in bovines. TNP2 contains two introns and an exon, and it encodes 132 amino acids. The TNP2 intron splits the first and second bases of a codon. The codon contains GT–AG splice sites (not shown) that comprise the vast majority of mammalian splice junctions. A previous study demonstrated that TNP2 mutations affect sperm chromatin structure and reduce fertility in mice [28]. In the present study, we discovered three SNPs within exon 1, intron 1, and the 3′-UTR region of the bovine TNP2 gene. The SNPs were found by examining 392 Chinese Holstein bulls via PCR–RFLP, CRS–PCR, and DNA sequencing.

Association analysis showed that the three SNPs markers g.269 G>A, g.480 C>T, and g.1536 C>T are significantly correlated with sperm quality traits excluding the environment and peculiarities in the AI stations. We identified SNP g.269 G>A as the possible causative polymorphism locus because it changes codon 62 of TNP2 from Arg to His. TNP2 is a 14 kDa protein with distinct structural domains [29]. TNP2 also has a very basic carboxyl terminal domain. The carboxyl terminal region of the molecule is rich in basic residues and a possible major site for electrostatic DNA binding. By contrast, the amino terminal region has two proposed zinc fingers [30]. The preferential binding activity of TNP2 to CpG sequences is often associated with promoter regions and dependent on zinc in rats [31]. In the g.269 G>A locus located in the middle region of the protein, the hydrophobicity and antigenic index of the protein changed (Fig. 2) when the amino acid changed. Hence, the non-synonymous SNP g.269 G>A may cause changes in the secondary structure of TNP2, leading to the changes in protein function.

The results also revealed a TNP2 SNP at position g.480 C>T in the intron that was significantly correlated with the sperm quality traits. Although introns do not code for proteins, they have important regulatory roles in gene expression and regulation [32] and mRNA transcription and splicing [33]. We need to clarify whether the g.480 C>T mutation affects gene expression and regulation.

Bulls with genotype CT at locus g.1536 C>T (3′-UTR) had higher ejaculate volumes and post-thaw cryopreserved sperm motility than those with genotype CC. Furthermore, the expression of TNP2 mRNA with genotypes CT and CC was significantly higher than those with genotype TT at locus g.1536 C>T (3′-UTR). Finally, bta-miR-154 exerted its inhibitory effects through the direct binding of the TNP2 3′-UTR with g.1536 C>T-T, whereas TNP2 binding was not observed in the g.1536 C>T-C SNP mutation. Our findings suggest that SNPs influence TNP2 gene expression by modulating the binding affinities of the miRNA and the target mRNA. The SNP may thus be responsible for the differences in semen quality of bulls. Polymorphisms and mutations in the corresponding sequences, such as the machinery, miRNA precursors, and target sites, are likely to significantly contribute to phenotypic variations such as disease susceptibility [34]. Genome-wide bioinformatics analysis predicted that approximately 64% of the transcribed target SNPs increase or decrease the binding energy of putative miRNA–mRNA duplexes by more than 90% [17]. Several studies have reported that the posttranscriptional gene regulation mediated by microRNA plays an important role in the development of male reproductive organs and germ cells in mammals. Using software such as TargetScan, RNA22, and RNA hybrid prediction, we found that the g.1536 C>T locus is located in the 3′-UTR of the TNP2 gene. We also found a conserved region that matches the miR-154 seed region in the mutant genotype TT. However, the CC genotype does not combine with miRNA-154. The SNP g.1536 C>T-C in the 3′-UTR region abolished the imperfect complementary binding of the bta-miR-154 seed sequence and the TNP2 gene, which increases TNP2 mRNA expression. The relative TNP2 mRNA expression in Holstein bulls with different genotypes was also consistent the aforementioned results. Furthermore, the luciferase reporter assay confirmed that the TNP2 expression was significantly downregulated in the TT genotype samples compared with the CC genotype samples. The low TNP2 expression may prevent spermatogenesis and further influence semen quality. These results provide the first indication that bta-miR-154 participates in the regulation of TNP2 expression and is further involved in determining the semen quality of bulls.

In summary, the g.1536 C>T in TNP2 is a functional candidate SNP that interacts with the miRNA target site. The g.1536 C>T in TNP2 has strong biological relevance and is likely involved in spermatogenesis. This research is promising because it shows an effective means of marker-assisted selection for semen quality and fertility of mammals. However, larger sample sets are still needed to confirm the association and for determining the possible effects of the variant on semen quality.
